# TROAP switches DYRK1 activity to drive hepatocellular carcinoma progression

**DOI:** 10.1038/s41419-021-03422-3

**Published:** 2021-01-26

**Authors:** Lei Li, Jia-Ru Wei, Ye Song, Shuo Fang, Yanyu Du, Zhuo Li, Ting-Ting Zeng, Ying-Hui Zhu, Yan Li, Xin-Yuan Guan

**Affiliations:** 1 State Key Laboratory of Oncology in South China and Collaborative Innovation Center for Cancer Medicine, Sun Yat-sen University Cancer Center, 510060 Guangzhou, China; 2 Department of Clinical Oncology, State Key Laboratory for Liver Research, The University of Hong Kong, Hong Kong, China; 3 Department of Clinical Oncology Center, The University of Hongkong-Shenzhen Hospital, 518053 Shenzhen, China; 4 State Key Laboratory of Ophthalmology, Zhongshan Ophthalmic Center, Sun Yat-sen University, 510060 Guangzhou, China; 5 Affiliated Cancer Hospital & Institutes of Guangzhou Medical University, Guangzhou Key Medical Discipline Construction Project, 510095 Guangzhou, China; 6 The Seventh Affiliated Hospital, Sun Yat-sen University, 518100 Shenzhen, China

**Keywords:** Oncogenes, Cell growth

## Abstract

Hepatocellular carcinoma (HCC) is one of the common malignancy and lacks effective therapeutic targets. Here, we demonstrated that ectopic expression of trophinin-associated protein (TROAP) dramatically drove HCC cell growth assessed by foci formation in monolayer culture, colony formation in soft agar and orthotopic liver transplantation in nude mice. Inversely, silencing TROAP expression with short-hairpin RNA attenuated the malignant proliferation of HCC cells in vitro and in vivo. Next, mechanistic investigation revealed that TROAP directly bound to dual specificity tyrosine phosphorylation regulated kinase 1A/B (DYRK1A/B), resulting in the cytoplasmic retention of proteins DYRK1A/B and promoting cell cycle process via activation of Akt/GSK-3β signaling. Combination of cisplatin with an inhibitor of DYRK1 AZ191 effectively inhibited tumor growth in mouse model for HCC cells with high level of TROAP. Clinically, TROAP was significantly upregulated by miR-142-5p in HCC tissues, which predicted the poor survival of patients with HCC. Therefore, TROAP/DYRK1/Akt axis may be a promising therapeutic target and prognostic indicator for patients with HCC.

## Introduction

Hepatocellular carcinoma (HCC) is the most common pathological type of liver cancer, accounting for 75%–85% of cases^1^. Owing to the property of malignant proliferation, HCC is one of the most aggressive malignancies without effective targeted therapies. Therefore, investigation of molecular mechanisms underlying HCC malignant progression and identification of effective therapeutic targets are urgently needed for improving outcomes of patients with HCC.

Trophinin-associated protein (TROAP), also known as tastin, was first cloned from human epithelial cells in 1995^2^. It as a cytoplasmic protein is composed of 778 amino acid residues and contains potential phosphorylation sites for protein kinases. TROAP highly expresses in bone marrow, testis and thymus, and is involved in centrosome integrity and spindle assembly during mitosis. During embryo implantation, TROAP can form an adhesion molecule complex with bystin and trophinin facilitating cell adhesion^3,4^. In addition, TROAP also plays an essential role in cell proliferation. However, its biological functions in cancer remain to be elucidated. Recent studies have gradually revealed the oncogenic role of TROAP in several digestive system malignancies, such as prostate cancer, gastric cancer, colorectal cancer, and HCC^5–9^. TROAP was upregulated in prostate cancer tissues and predicted the poor survival of prostate cancer patients^5^. TROAP played an oncogenic role in gastric cancer by affecting cell proliferation and invasion^6^. Using Oncomine database analysis, *TROAP* was confirmed to be upregulated in human colorectal cancer tissues^7^. Moreover, the expression and functions of TROAP in HCC have been superficially explored by several research groups. TROAP expression was significantly increased in HCC tissues compared with adjacent non-tumor liver tissues. The Cancer Genome Atlas (TCGA) database analysis showed that *TROAP* might serve as an independent prognostic factor for poor survival in HCC patients^8,9^. TROAP expression was increased during the G2/M phase and abruptly declined after the cell division, suggested that TROAP was involved in cell proliferation^8^. Inversely, one study has proved that TROAP played an inhibitory role on tumor growth and metastasis in HCC^10^. The inconsistent functions of TROAP in HCC progression were still undetermined. Therefore, the expression regulation, functional mechanism, and therapeutic potential of TROAP in HCC needed to be revealed.

In the present study, we illuminated that the mRNA expression of *TROAP* was specifically regulated by *miR-142-5p* in HCC. Upregulated TROAP dramatically enhanced HCC cell proliferation via direct interaction with dual specificity tyrosine phosphorylation regulated kinase 1 A/B (DYRK1A/B). Mechanically, TROAP increased the cytoplasmic levels of DYRK1A/B and activated Akt/GSK-3β signaling. Importantly, an inhibitor of DYRK1A/B AZ191 could inhibit tumor growth in mice for HCC cells with high level of TROAP. Therefore, targeting DYRK1A/B may be a promising therapeutic strategy for HCC patients with high expression of TROAP.

## Materials/subjects and methods

### Clinical samples and cell lines

Primary HCC and non-tumor liver tissues were collected from the Sun Yat-sen University Cancer Center (Guangzhou, China). Written informed consents were obtained from all recruited patients before samples were collected. All clinical samples used in this study were approved by the Committees for Ethical Review at the Sun Yat-sen University Cancer Center (Guangzhou, China). Human HCC cell lines Huh7, HepG2, Hep3B and PLC8024 were purchased from the American Type Culture Collection (ATCC, Manassas, VA). Human immortalized hepatocyte MIHA was provided by Dr. J. R. Chowdhury (Albert Einstein College of Medicine, NY). All cell lines have been authenticated by STR profiling and tested for mycoplasma contamination. All cells were cultured with high-glucose Dulbecco’s modified Eagle medium (DMEM; Gibco, Grand Island, NY) supplemented with 10% fetal bovine serum (FBS, Gibco, Grand Island, NY) at 37 ˚C with 5% CO_2_.

### Transfection of plasmids and oligonucleotides

For exogenous overexpression of *TROAP*, the coding sequence of human *TROAP* was cloned into lentiviral expression vector LV242 (GeneCopoeia, Rockville, MD). One short-hairpin RNA (shRNA) targeting *TROAP* (sh*TROAP*, Table S1) was cloned into lentiviral interference vector psi-LVRU6GP (GeneCopoeia, Rockville, MD). For lentivirus package, recombinant plasmid and three lentivirus packaging vectors, including pLp1, pLp2 and pLp-VSVG (Invitrogen, Carlsbad, CA), were co-transfected into 293FT cells (Invitrogen, Carlsbad, CA) using HilyMax transfection reagent (#H357, Dojindo, Japan). Empty vector LV242 and psi-LVRU6P containing scrambled shRNA were also transfected as a negative control, respectively. Culture supernatant with lentivirus was harvested to infect HCC cells. Stable cell clones were selected by Puromycin (Sigma, Burlington, MA) treatment. In addition, small interfering RNA specifically targeting *DYRK1A* or *DYRK1B* (Table S1) was transfected into Huh7 cells using HilyMax reagent to transiently silence *DYRK1*. To explore the regulation of *miR-142-5p* in the expression of *TROAP*, human *miR-142-5p* mimics and negative control oligonucleotides (Table S1) were also transfected with HilyMax reagent. Next, 48 h after transfection, the mRNA expressions of genes were analyzed with quantitative real-time PCR (qRT-PCR).

### RNA isolation and qRT-PCR

Human fresh HCC tissues were lysed with TRIzol™ Reagent (#15596026, Invitrogen, Carlsbad, CA) after homogenate using a homogenizer. For HCC cells, TRIzol Reagent was directly added to the culture dish to lyse the cells after remove of growth media. RNA isolation was strictly performed according to the procedural guidelines of the reagent (Pub. No. MAN0001271, Invitrogen, Carlsbad, CA). Complementary DNA (cDNA) was synthesized through reverse transcription using PrimeScript™ RT Reagent Kit with gDNA Eraser (#RR047A, Takara, Japan). Next, qRT-PCR was carried out using FastStart Universal SYBR Green Master (#4913914001, Roche, Switzerland, Basel) and a Real-Time PCR Detection System (Roche, Switzerland, Basel). The expressions of *ACTB* and *U6* were also analyzed as internal control. The gene-specific primers used were listed in Table S1. The relative expressions (defined as fold change) of the target genes (2^−ΔΔCt^) were normalized to the endogenous *ACTB* or *U6* references (ΔCt).

### Immunostaining

For immunohistochemistry (IHC) staining in paraffin-embedded tissues was performed as described previously^11^. In brief, sections were deparaffinized in pure xylene for three times (15 min per time) and rehydrated with a concentration gradient of alcohol (100%, 95%, 75% and 50%, 5 min per time). Next, slides were incubated with 3% hydrogen peroxide at room temperature for 15 min to inactivate endogenous peroxidase. For antigen retrieval, tissues sections were boiled in 1× EDTA Antigen Retrieval Solution (#P0085, Beyotime, China) for 15 min in electric pressure cooker. Nonspecific binding was blocked with 5% bull serum albumin (BSA, Amresco, Boise, ID) at 37 °C for 30 min. Primary antibodies against TROAP (#SC271716, Santa Cruz Biotechnology, 1:100 dilution), Ki67 (#ab16667, Abcam, 1:400 dilution), DYRK1A (#8765, Cell Signaling Technology, 1:100 dilution) and DYRK1B (#5672, Cell Signaling Technology, 1:100 dilution) were incubated at 4 °C overnight in a humidified chamber. After wash with phosphate buffer saline (PBS) three times (5 min per times), the slides were detected using HRP-conjugated secondary antibody and DAB substrate system (#K346711-2, Dako, Santa Clara, CA). Cell nuclei were counterstained with hematoxylin (#TA-125-MH, Thermo Fisher Scientific, Waltham, MA) before imaging by microscope (Olympus, Lake Success, NY). For immunofluorescent (IF) staining in adherent cells, growing cells on chamber slides were fixed with 4% paraformaldehyde (#P0099, Beyotime, China) and treated with 0.1% Triton X-100 (#ST795, Beyotime, China) for cell permeabilization. After blocking with 5% BSA, cell slides were incubated with the primary antibodies as mentioned above at 4 °C overnight in a moist chamber. After thorough wash with PBS, the slides were then incubated with Alexa Fluor® 594 or 488 conjugated secondary antibodies (Invitrogen, Carlsbad, CA). Finally, all slides were mounted with Mounting Medium with DAPI (#ab104139, Abcam, Cambridge, MA) and imaged by an OLYMPUS FV2000 fluorescence microscope.

### Protein extraction and western blot analysis

Cell total proteins were extracted using cold 1× RIPA buffer (#9806, Cell Signaling Technology, Danvers, MA) supplemented with protease inhibitor cocktail (#4693159001, Roche, Switzerland, Basel) and phosphatase inhibitor PhosSTOP (#490683700, Roche, Switzerland, Basel). NE-PER™ Nuclear and Cytoplasmic Extraction Reagents (#78833, Thermo Fisher Scientific, Waltham, MA) were used to separate the cytoplasmic and nuclear proteins of HCC cells according to the user instructions. Western blotting was performed as described previously^12^. The specific primary antibodies were listed in Table S2. Relative expressions of proteins were analyzed with ImageJ software (https://imagej.nih.gov/ij/).

### Cell cycle distribution analysis

HCC cells were pre-fixed with 70% cold alcohol at 4 °C overnight. The cells were washed twice with PBS (×1000 rpm, 5 min per time) and stained with 50 μg/ml Propidium Iodide (# P4170, Sigma, Burlington, MA) supplemented with 100 μg/ml RNase A (#R6513, Sigma, Burlington, MA) and 0.1% Triton X-100 (#ST795, Beyotime, China) at 37 °C for 30 min. Cell cycle distributions were analyzed by FACS system (Beckman Coulter, Boulevard Brea, CA) and ImageJ software (https://imagej.nih.gov/ij/).

### In vitro cell proliferation assays

Cell proliferation was analyzed by cell growth curves, foci formation in monolayer culture, and colony spheres formation in soft agar assays. First, cells were seeded into 96-well plates (1000 cells per well) and cell growth rates were measured every day for four time using a Cell Counting Kit-8 (#CK04, Dojindo, Japan) according to the user instructions. For anchorage-dependent foci formation assay, 2000 cells were planted in 6-well plates, or 300 cells were seeded into 24-well plates. After one week of culture at 37 ˚C with 5% CO_2_, plates were stained with Crystal Violet Staining Solution (#C0121, Beyotime, China), and the numbers of cell foci were counted with Alpha FluorChem SP software. Colony formation frequency in soft agar was used to assess the anchorage-independent growth of HCC cells. In brief, 5000 cells were suspended in soft agar mixture (DMEM, 10% FBS and 0.4% Sea Plaque agarose) and were subsequently overlaid on the solidified 0.6% agar base. Cells were cultured for one week at 37 ˚C with 5% CO_2_. Spheres were counted and imaged using a microscope (Olympus, Lake Success, NY).

### In vivo xenograft tumor assay

All animal experiments were approved by Animal Ethics Committee at Sun Yat-sen University Cancer Center (Guangzhou China). Four-week-old male BALB/c nude mice were purchased from the Guangdong Medical Laboratory Animal Center (Guangzhou China). Mice of the same age and sex were randomly assigned to experimental groups. For subcutaneous xenograft tumor model, cell suspensions (2 × 10^6^ Hep3B or 2 × 10^6^ PLC8024 per mouse) in 100 μl PBS were subcutaneously injected into nude mice. Three weeks after injection, tumor weights were measured with electronic scales after sacrifice of mice with euthanasia. Tumor volumes were calculated as volume (mm^3^) = length × width^2^ × 0.5. For orthotopic liver xenograft tumor model, preventive analgesic with buprenorphine was provided before surgery. Mice were anesthetized with Pentobarbitone (80 mg/kg body weight, *i.p*.), and the abdomen was sterilized with iodine and alcohol swabs. An incision (about 5–8 mm) was made through the middle upper abdomen. The hepatic lobe was carefully exposed with sterile cotton swab. Cell suspensions (1 × 10^6^ HepG2 or 1 × 10^6^ Huh7 per mouse) in 40 μl PBS and Matrigel (v/v = 1:1) were injected into liver with sterile insulin syringes. The hepatic lobe was then returned to the peritoneal cavity. The abdominal wall was closed with 4.0 Dexon sutures and skin was closed with Monofilament Nylon. Three weeks after cell injection, all of mice were euthanized. Tumor nodules in the liver were detected under light microscope after hematoxylin-eosin staining.

### Statistical analysis

SPSS version 17.0 (Chicago, IL) and GraphPad Prism 5 (San Diego, CA) were used for data analyses. Two-sided independent Student’s *t*-test was used for continuous data between two groups. Gene expression levels and survival curves in TCGA database were directly produced from GEPIA 2 (http://gepia2.cancer-pku.cn/)^13^. Genes Co-expression, Gene Ontology and Disease Ontology analyses were performed using Coexpedia (http://www.coexpedia.org/)^14^. Proteins that potentially bound to TROAP were obtained from a protein-protein interaction database InAct (http://www.ebi.ac.uk/intact/)^15^. If the *P*-value is less than 0.05, results were statistically significant.

## Results

### TROAP accelerates cell cycle process in HCC cells

**Fig. 1:**
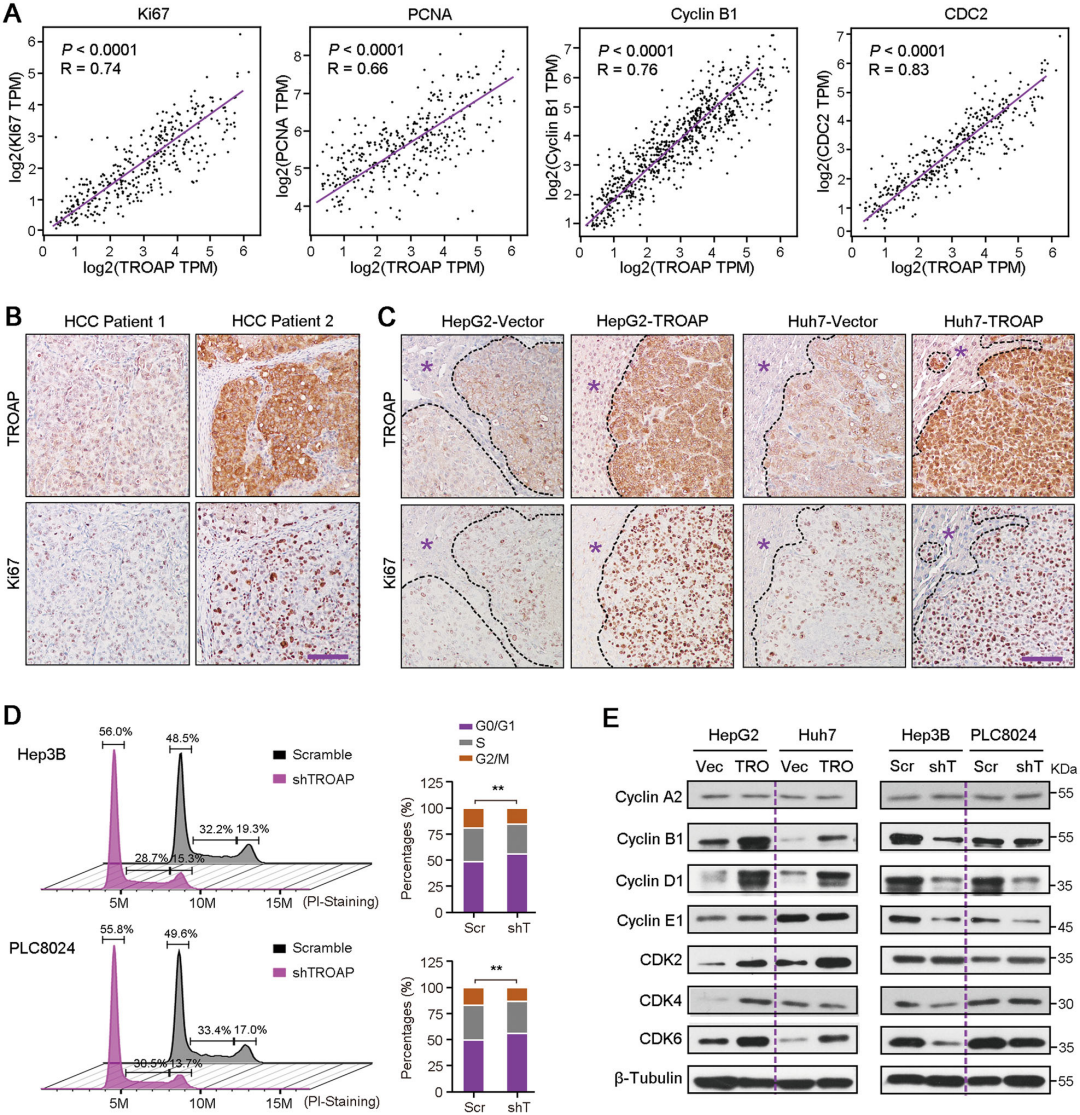
TROAP enhances HCC cell growth by accelerating cell cycle process. **A** The mRNA expression correlations between TROAP and cell proliferation-associated genes in HCC tissues were analyzed using TCGA database. IHC staining with antibodies against TROAP and Ki67 were performed in serial sections from two HCC patients with low or high expression of TROAP (**B**), or from orthotopic xenograft tumors derived from vector or TROAP-transfected HepG2 and Huh7 cells (**C**), respectively. Scale bar, 200 μm. In **C**, normal liver tissues were indicated by asterisks. **D** The cell cycle distributions of Hep3B and PLC8024 cells with or without TROAP silence were analyzed by flow cytometry. One way ANOVA; **P < 0.01. **E** Western blotting was used to analyze the expressions of cell cycle-associated proteins in HepG2 and Huh7 cells transfected with vector or TROAP, and in Hep3B and PLC8024 cells with or without TROAP knockdown. β-Tubulin was also tested as a loading control.

Correlation analysis of mRNA expressions in TCGA cohorts showed that *TROAP* expression was significantly and positively correlated with the levels of genes that involved in cell proliferation and cell cycle, such as *Ki67*, *PCNA*, *Cyclin B1* and *CDC2* (*P* < 0.001, Fig. 1A). IHC staining in serial sections also confirmed a positive correlation between expressions of TROAP and Ki67 in human HCC tissues (Fig. 1B) and xenograft tumors derived from HepG2 and Huh7 cells with or without *TROAP* overexpression (Fig. 1C). These data suggest the key role of TROAP in HCC proliferation. In addition, cell cycle distribution was analyzed with flow cytometry assay, and results showed that silence of *TROAP* slowed the cell cycle of Hep3B and PLC8024 cells (Fig. 1D). Western blotting showed the increased expressions of Cyclin B1, Cyclin D1, CDK2 and CDK6 in *TROAP*-overexpressed HepG2 and Huh7 cells (Fig. 1E, left). Inversely, knockdown of *TROAP* resulted in the downregulation of these cell cycle-related proteins in *TROAP*-silenced Hep3B and PLC8024 cells (Fig. 1E, right). These findings suggest the pro-proliferation activity of TROAP in HCC cells.

### Exogenous overexpression of TROAP drives HCC cell growth

**Fig. 2:**
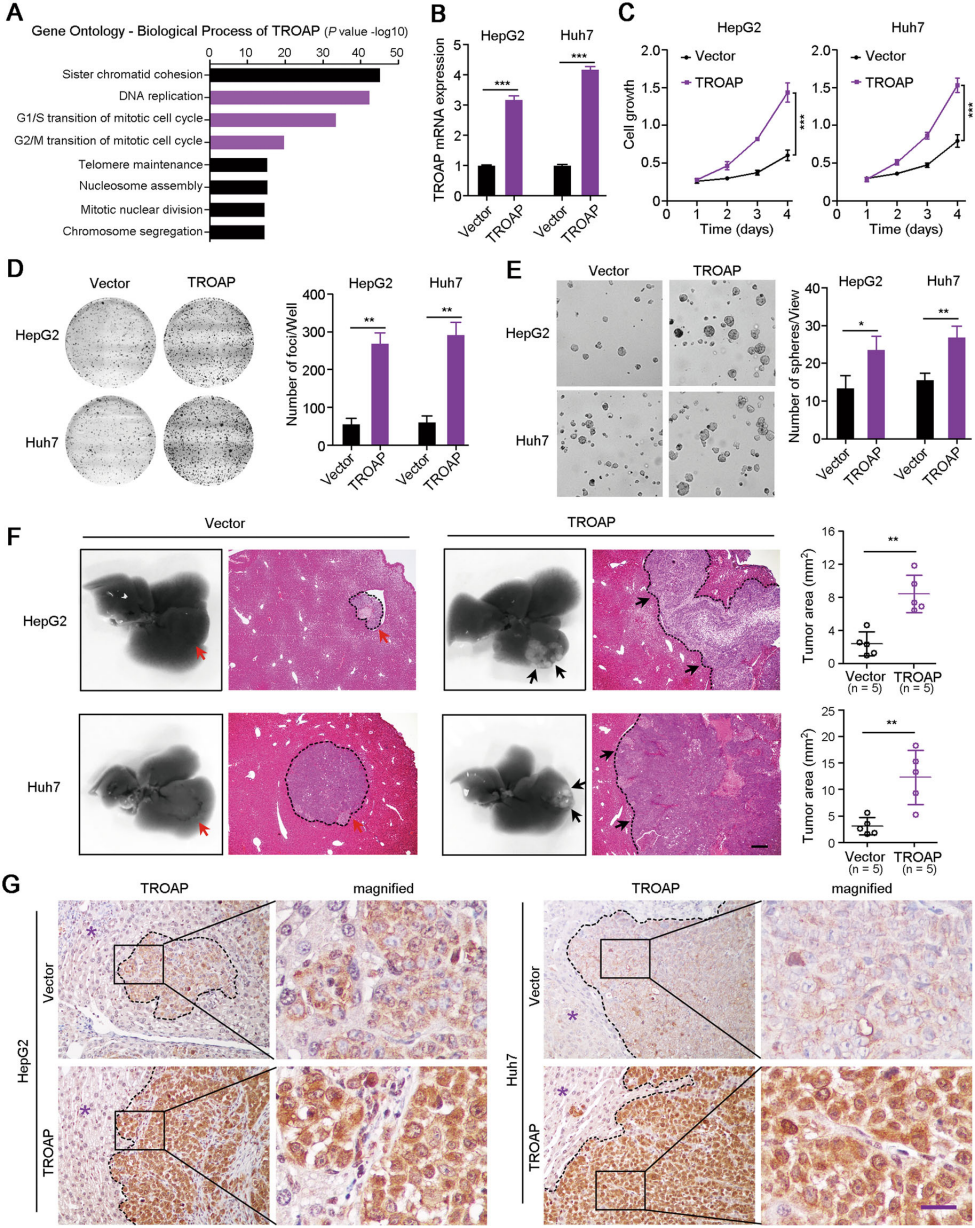
Overexpression of TROAP promotes HCC cell growth in vitro and in vivo. **A** Gene Ontology (biological process) analysis of TROAP in human using Coexpedia internet tool (http://www.coexpedia.org/). **B** Overexpression of TROAP was confirmed with RT-qRCR assay in HepG2 and Huh7 cells after lentivirus-mediated transfection of TROAP. Cell growth curves (**C**), foci formation (**D**), and sphere formation (**E**) assays were used to investigate the activity of TROAP in HCC cells, respectively. **F** Orhtotopic liver transplantation of HCC cells after overexpression of TROAP in nude mice. Xenograft nodes were indicated by arrows. Tumor area was analyzed with ImageJ software and summarized in right panel. **G** IHC staining confirmed the high expression of TROAP in xenograft tumors derived from TROAP-overexpressed HCC cells. Asterisk, normal liver tissue; Scale bar, 50 μm. In panels **B**–**F**, data are represented as mean ± SD; two-sided Student’s *t*-test; *P < 0.05; **P < 0.01; ***P < 0.001.

Gene Ontology (biological process) analysis showed that *TROAP* was closely related to chromosome assembly and segregation, DNA replication and cell cycle regulation (Fig. 2A). Previous studies^5,6^ and our IF staining indicated the high expression of *TROAP* during mitotic phase in HCC cells (Fig. S1). Herein, to confirm the roles of *TROAP* in HCC cell growth, HepG2 and Huh7 cells with relative low expression of *TROAP* were transfected with lentivirus containing the coding sequence of *TROAP* or empty vector (Fig. 2B). Cell growth curves showed that *TROAP*-transfected cells proliferated faster than control cells (*P* < 0.001, Fig. 2C). Moreover, adherent foci formation in monolayer culture (*P* < 0.01, Fig. 2D) and non-adherent colony formation in soft agar (*P* < 0.05, Fig. 2E) assays also found the increased number of colonies of HepG2 and Huh7 cells after transfection of *TROAP*. Orthotopic liver injections of HCC cells in nude mice showed the larger tumor burden of mice injected with *TROAP*-overexpressed cells, indicating the tumor growth promotion of *TROAP* (*P* < 0.01, Fig. 2F). IHC staining confirmed the higher expressions of protein TROAP in xenograft tumors derived from *TROAP*-transfected cells than tumors from control cells (Fig. 2G).

### TROAP silence attenuates the malignant proliferation of HCC cells

**Fig. 3:**
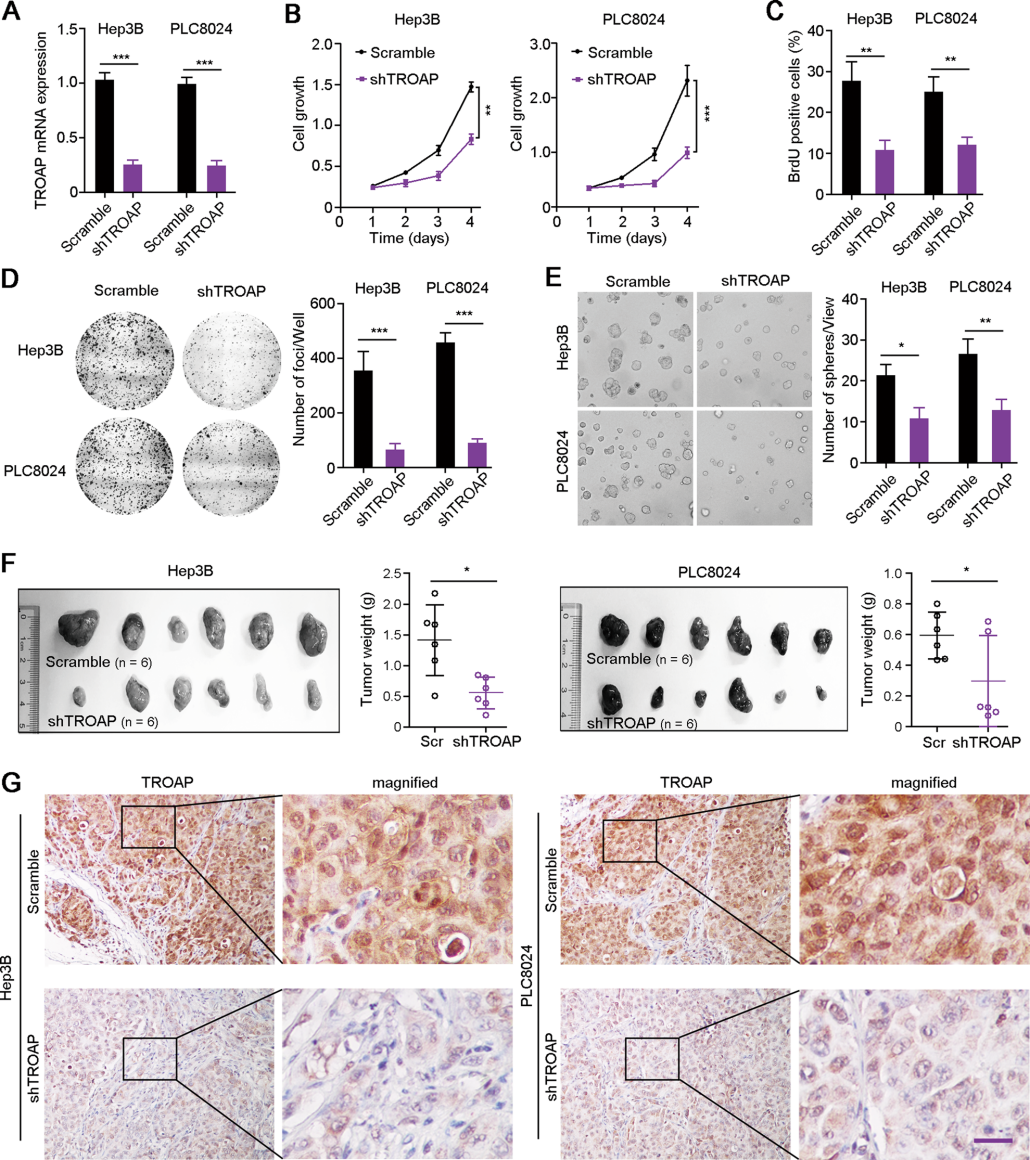
Downregulation of TROAP attenuates the malignant proliferation of HCC cells in vitro and in vivo. **A** The downregulation of TROAP in Hep3B and PLC8024 cells after lentivirus-mediated silence of TROAP was confirmed by qRT-PCR. Cell growth (**B**), BrdU incorporation (**C**), foci formation (**D**), and sphere formation (**E**) assays were used to analyze HCC cell proliferation after knockdown of TROAP. Scramble vector was also transfected as a control. **F** Hep3B and PLC8024 cells transfected with shTROAP or scramble control were subcutaneously injected into nude mice. Three weeks after injection, tumor weights were measured. **G** IHC staining confirmed the knockdown of TROAP in xenograft tumors derived from TROAP-silenced HCC cells. Scale bar, 50 μm. In panels **B**–**F**, data are indicated as mean ± SD; two-sided Student’s *t*-test; *P < 0.05; **P < 0.01; ***P < 0.001.

To further investigate the aggressive role of *TROAP*, one shRNA targeting *TROAP* (sh*TROAP*) was stably transfected into Hep3B and PLC8024 cells that highly expressed *TROAP* (Fig. 3A). Cell growth (*P* < 0.01, Fig. 3B) and BrdU incorporation (*P* < 0.01, Fig. 3C) assays demonstrated the decreased proliferation rate of Hep3B and PLC8024 cells after knockdown of *TROAP* expression. Colony formation assays revealed that silence of *TROAP* in HCC cells reduced the frequency of foci formation in monolayer culture (*P* < 0.001, Fig. 3D) and spheres formation in soft agar (*P* < 0.05, Fig. 3E). Moreover, scramble vector or sh*TROAP*-transfected HCC cells were subcutaneously injected into nude mice, and results showed that the weight of xenograft tumors derived from TROAP-silenced cells was significantly lighter than tumors developed from control cells (*P* < 0.05, Fig. 3F). In addition, decreased expression of protein TROAP in xenograft tumors derived from sh*TROAP*-treated cells were detected by IHC staining (Fig. 3G). These in vitro and in vivo functional assays demonstrate that high expression of TROAP drives the malignant proliferation of HCC cells.

### DYRK1A and DYRK1B mediate the proliferation promotion of TROAP

**Fig. 4:**
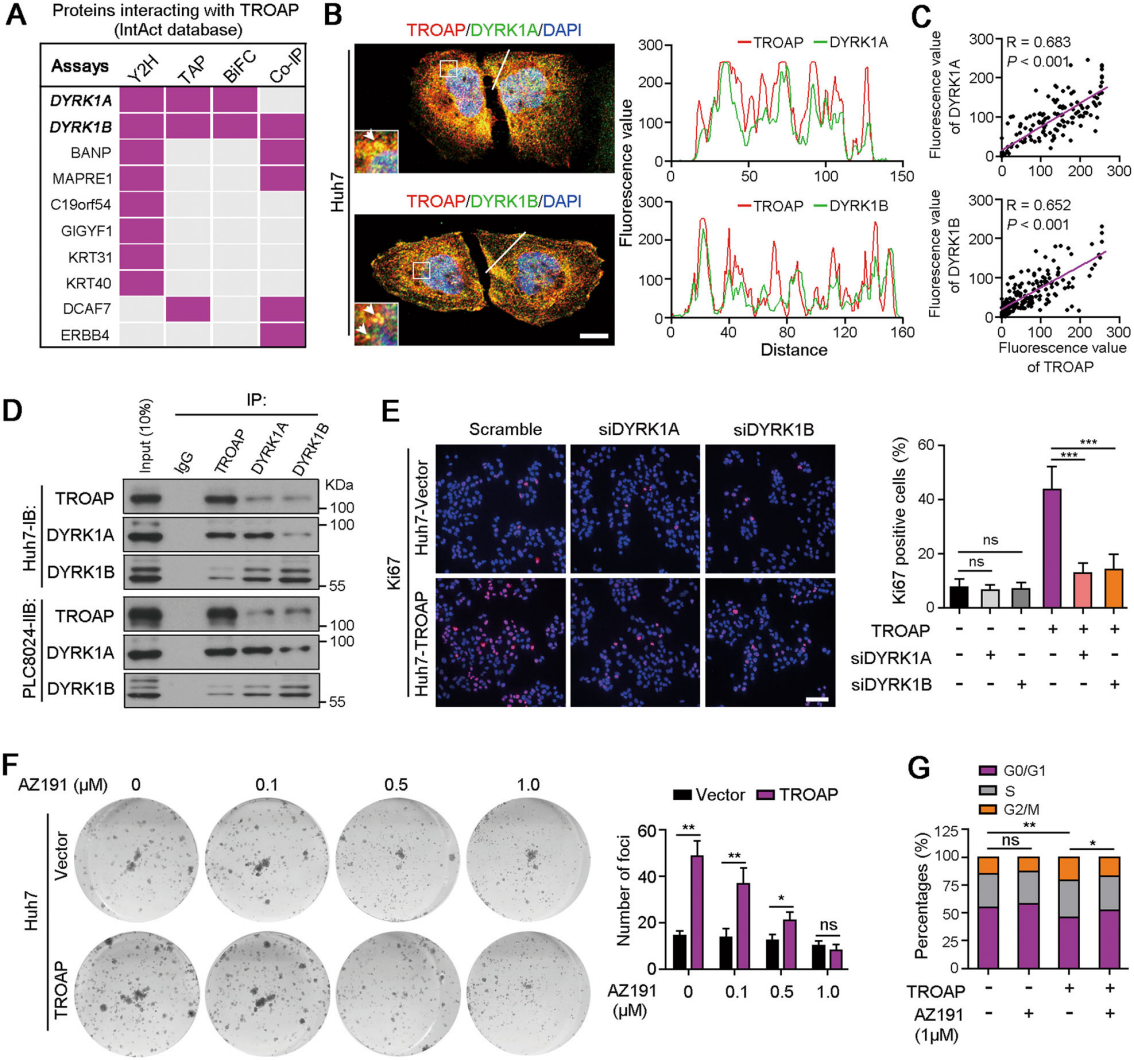
TROAP interacts with DYRK1A and DYRK1B promoting HCC cell growth. **A** IntAct database (https://www.ebi.ac.uk/intact/) analysis showed TROAP interacting proteins that have been proved by different methods, such as yeast two-hybrid analysis (Y2H), tandem affinity purification (TAP), bimolecular fluorescence complementation (BiFC) and co-immunoprecipitation (Co-IP). **B** Immunofluorescent (IF) double-staining with antibodies against TROAP (red) and DYRK1A or DYRK1B (green) in Huh7 cells. Cell nuclei were stained with DAPI (blue). Scale bar, 10 μm. Representative co-localization signals indicated by white line were analyzed with ImageJ software. **C** The correlation of fluorescence values between TROAP and DYRK1A or DYRK1B were analyzed, respectively. **D** Co-IP analysis was performed to confirm the directly interaction of TROAP with DYRK1A or DYRK1B in Huh7 and PCL8024 cells. **E** IF staining with antibody against Ki67 (a marker of proliferative cell) in vector or TROAP-transfected Huh7 cells with or without DYRK1A/B silence. Scale bar, 100 μm. The percentages of Ki67 positive cells were summarized in right panel. **F** Foci formation analysis of vector or TROAP-transfected Huh7 cells under treatments with different concentration of DYRK1A/B inhibitor AZ191 (#S7338, Selleck). **G** The cell cycle distributions of Huh7 cells with or without TROAP overexpression or and AZ191 treatment (1 μM, 24 h) were analyzed by flow cytometry. In panels **E**, **F** data were indicated as mean ± SD; two-sided Student’s *t*-test; **G** One way ANOVA; *P < 0.05; **P < 0.01; ***P < 0.001; ns, no statistical significance.

By analyzing protein-protein interaction database InAct, we found that TROAP could directly bind to DYRK1 protein family, including DYRK1A and DYRK1B (Fig. 4A and Table S3). Double IF staining in Huh7 and PLC8024 cells displayed the co-localization of TROAP with DYRK1A or DYRK1B (Fig. 4B, C and Fig. S2). Protein co-immunoprecipitation (co-IP) assay demonstrated the interaction among these proteins in HCC cells (Fig. 4D). DYRK1 family members are protein phosphokinases involved in cancer progression by regulating cell proliferation^16,17^. To explore the roles of DYRK1A and DYRK1B in TROAP-mediated HCC growth, small interfering RNAs targeting *DYRK1A* or *DYRK1B* were transfected into Huh7 cells with or without *TROAP* overexpression (Fig. S3). Ki67 staining indicated that knockdown of *DYRK1A* or *DYRK1B* attenuated the pro-proliferation effect of *TROAP* (Fig. 4E). Moreover, a small-molecule inhibitor of DYRK1 AZ191 (1 μM) that could inhibit HCC cell growth was used to treat vector or *TROAP*-transfected Huh7 cells (Fig. 4F and Fig. S4). Foci formation (Fig. 4F) and cell cycle distribution (Fig. 4G) analyses showed that blocking DYRK1 with AZ191 effectively suppressed the cell proliferation of *TROPA*-overexpressed Huh7 cells.

### TROAP increases cytoplasmic DYRK1 to activate Akt/GSK-3β signaling

**Fig. 5:**
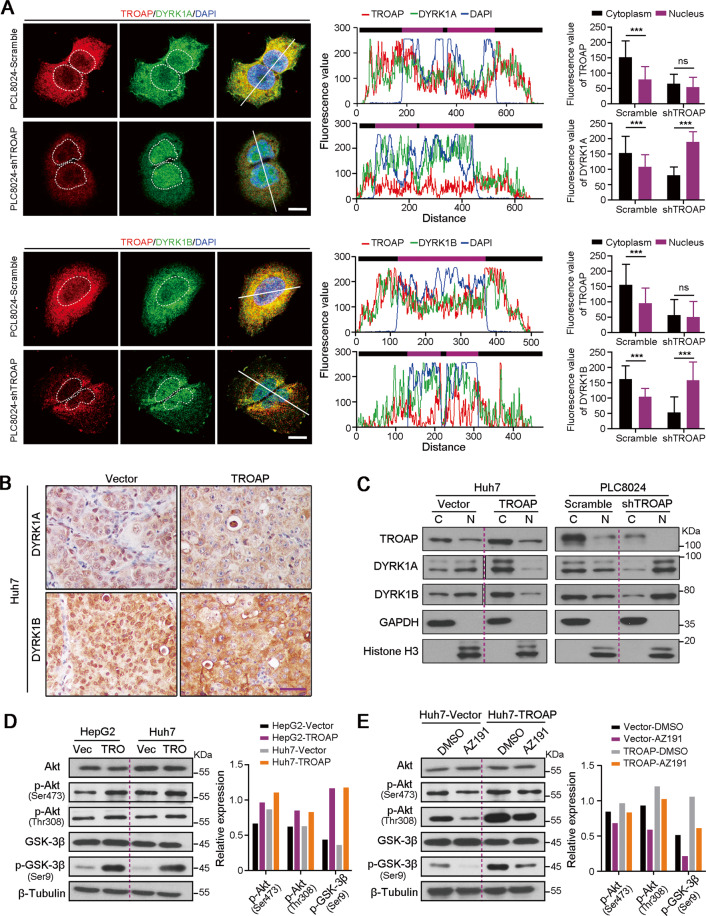
TROAP-mediated the cytoplasmic localization of DYRK1A and DYRK1B activates Akt/GSK-3β signaling. **A** IF double-staining with antibodies against TROAP (red) and DYRK1A or DYRK1B (green) in PLC8024 cells with or without TROAP silence. Cell nuclei were stained with DAPI (blue). Scale bar, 10 μm. Representative fluorescence signals in cytoplasm and nucleus indicated by white line were analyzed with ImageJ software and summarized in right panel. In right panel, data were indicated as mean ± SD; two-sided Student’s *t*-test; ***P < 0.001; ns, no statistical significance. **B** IHC staining with antibodies against DYRK1A and DYRK1B in xenograft tumors derived from vector or TROAP-transfected Huh7 cells. Scale bar, 50 μm. **C** The protein expressions of TROAP, DYRK1A, and DYRK1B in cytoplasm and nucleus in Huh7 cells transfected with vector or TROAP and PLC8024 cells with or without TROAP silence were analyzed with western blotting, respectively. GAPDH and Histone H3 were also severally tested as cytoplasmic and nuclear protein indicators. C, cytoplasm; N, nucleus. **D** The activation of Akt/GSK-3β signaling in HepG2 and Huh7 cells after transfection of vector (Vec) or TROAP (TRO) was analyzed by western blotting. **E** Western blot was used to analyze the activation of Akt/GSK-3β signaling in vector or TROAP-transfected Huh7 cells under treatments with DMSO or AZ191 (1 μM, 12 hours). In panels **D** and **F**, β-Tubulin was also tested as a loading control. Relative expressions of phosphorylated Akt and GSK-3β were analyzed with ImageJ software and summarized in right panel.

Next, double IF staining proved that knockdown of *TROAP* induced the nuclear localization of proteins DYRK1A and DYRK1B in PLC8024 cells (Fig. 5A). The cytoplasmic retentions of DYRK1A and DYRK1B in xenograft tumors derived from TROAP-transfected Huh7 cells were also observed by IHC staining, compared to control cells (Fig. 5B). Moreover, the protein levels of TROAP, DYRK1A, and DYRK1B in cytoplasm and nucleus in Huh7 cells transfected with vector or *TROAP* and PLC8024 cells with or without *TROAP* silence were analyzed with western blotting, respectively. Results showed that cytoplasmic DYRK1 was increased after overexpression of *TROAP* in Huh7 cells, which was inversely reduced when *TROAP* was silenced in PLC8024 cells (Fig. 5C). These findings suggest that aberrant expression of TROAP regulates the spatial localization of DYRK1 family proteins in HCC cells. Recent study reported that inhibition of DYRK1B resulted in the downregulation of Akt phosphorylation in human pancreatic and ovarian cancer cells^18^. Therefore, the activation of Akt/GSK-3β signaling in HepG2 and Huh7 cells after transfection of vector or *TROAP* was analyzed by western blotting. Results showed the increased phosphorylation levels of Akt at Ser473 and Thr308, as well as GSK-3β at Ser9 in *TROAP*-overexpressed HCC cells (Fig. 5D). Importantly, AZ191 treatment (1 μM, 12 h) could attenuate the activation of Akt/GSK-3β signaling induced by *TROAP* overexpression in Huh7 cells (Fig. 5E).

### Blocking DYRK1 inhibits TROAP-overexpressed HCC cell growth in vivo

**Fig. 6:**
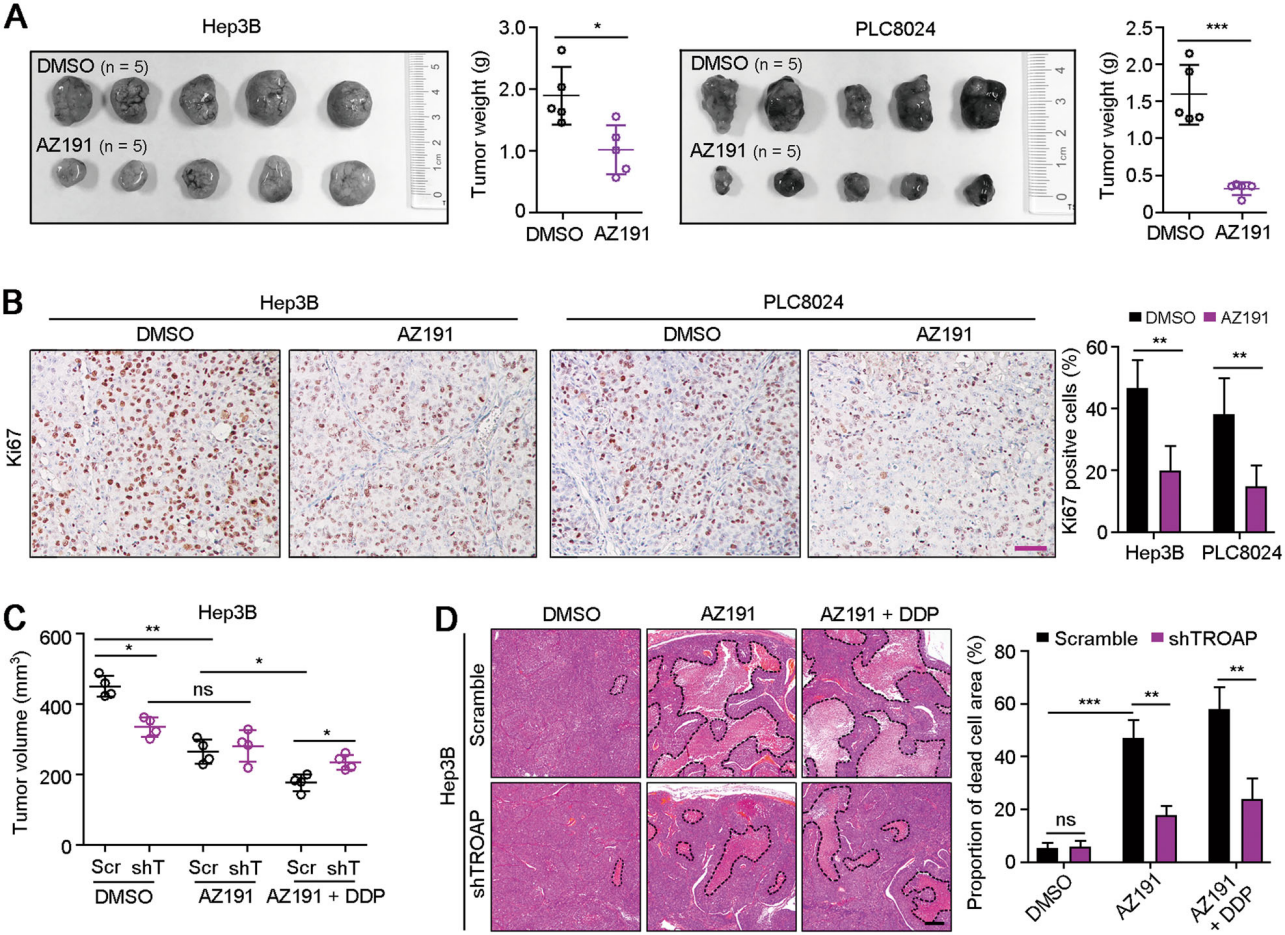
Combination of AZ191 and cisplatin suppresses HCC cell growth in nude mice. **A** Hep3B and PLC8024 cells were subcutaneously injected into nude mice. One week after injection, mice were treated with DMSO or AZ191 (50 mg/kg, *i.p*.) for six times, and tumor weights were measured after sacrifice of mice. **B** IHC staining with antibody against Ki67 in xenograft tumors treated with or without AZ191. The percentage of Ki67 positive cells were summarized in right panel. **C** Subcutaneous xenograft tumors derived from Hep3B cells transfected with scramble RNA (Scr) or shRNA targeting TROAP (shT) were treated with DMSO, AZ191 or/and cisplatin (DDP, 6 mg/kg, *i.p*.). Tumor volumes were measured after six times treatments. **D** H&E staining of xenograft tumors after treatments. The proportion of dead cell area was analyzed with ImageJ software and summarized in the right panel. In all panels, data were indicated as mean ± SD; two-sided Student’s *t*-test; *P < 0.05; **P < 0.01; ***P < 0.001; ns, no statistical significance.

To explore the targeted therapeutic potential of DYRK1 in HCC cells with upregulated *TROAP*, AZ191 was used to treat the subcutaneous xenograft tumors derived from Hep3B or PLC8024 cells in nude mice. Compared to DMSO treatment, tumor growth was significantly suppressed after six times injection of AZ191 (50 mg/kg, *i.p*.) (Fig. 6A). IHC staining also confirmed the lower percentage of Ki67 positive cells in xenograft tumors treated with AZ191 (Fig. 6B). In addition, subcutaneous xenograft tumors derived from Hep3B cells with or without *TROAP* silence were treated with DMSO, AZ191 (50 mg/kg, *i.p*.) or/and cisplatin (6 mg/kg, *i.p*.). Results showed that the growth inhibition of AZ191 in tumors with *TROAP* silence was weaker than that in control tumors, and of AZ191 in combination with cisplatin presented stronger inhibition of HCC cell growth in nude mice (Fig. 6C). H&E staining showed the greater proportion of dead cell area in xenograft tumors derived from scramble-transfected Hep3B cells after combination treatment, compared to *TROAP*-silenced tumors (Fig. 6D). Therefore, targeting *DYRK1* may be a promising therapeutic strategy for HCC patients with high expression of *TROAP*.

### TROAP is significantly upregulated and predicts the poor survival in HCC

**Fig. 7:**
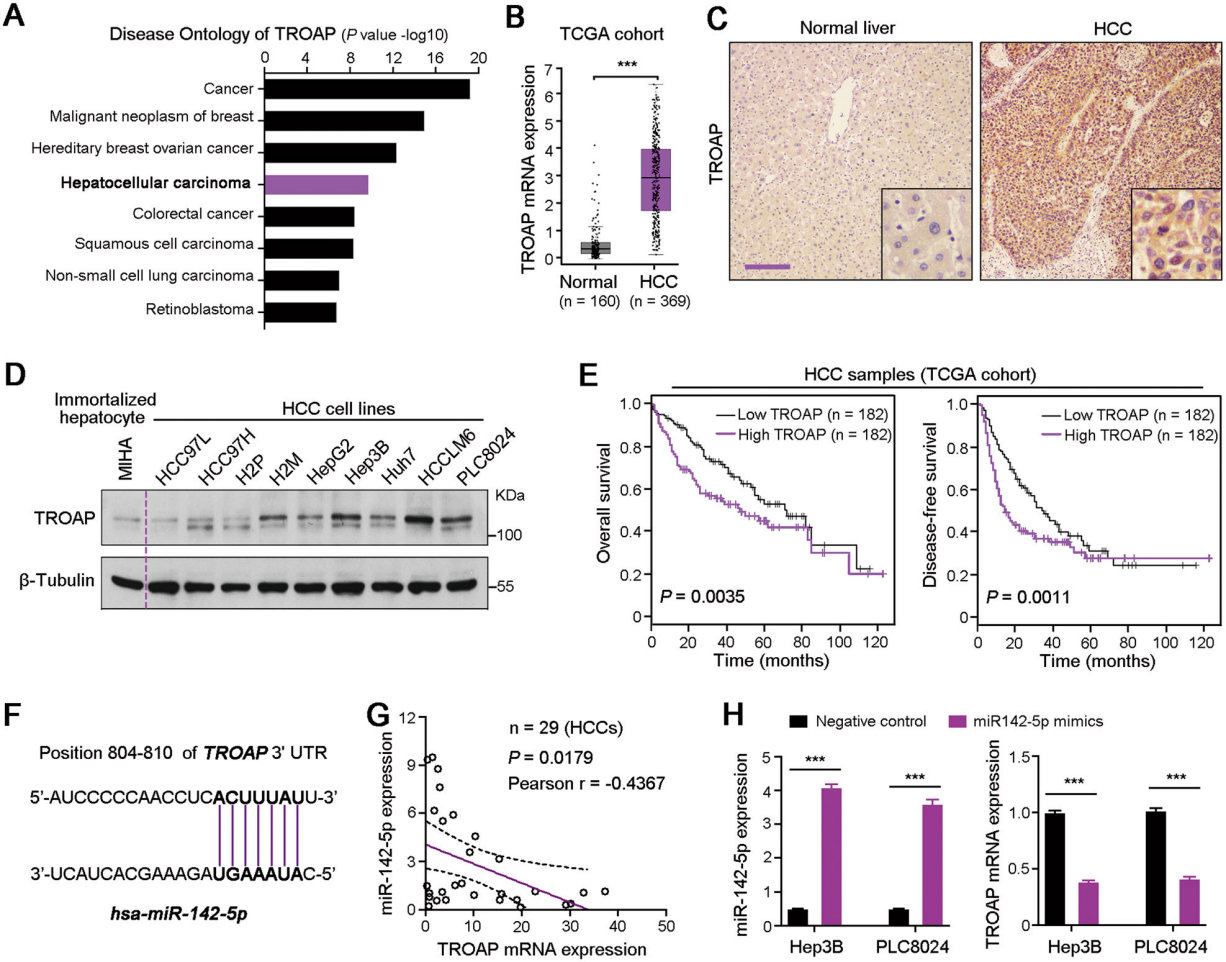
Upregulated TROAP predicts the poor outcome of HCC patients. **A** Disease Ontology analysis of TROAP in human using Coexpedia internet tool (http://www.coexpedia.org/). **B** TROAP mRNA expression level in normal liver and HCC tissues was analyzed based on The Cancer Genome Atlas (TCGA) cohort. Two-sided Student’s *t*-test; ***P < 0.001. **C** IHC staining of TROAP in paired normal liver and HCC tissues. Scale bar, 200 μm. **D** Western blotting analysis of TROAP expression in one immortalized liver cell line MIHA and nine HCC cell lines. β-Tubulin was used a loading control. **E** Kaplan–Meier survival curves based on TCGA database suggested that both overall survival and disease-free survival of the HCC patients with high TROAP expression were significantly shorter than those with low level of TROAP (Log-Rank test). **F** In human, miR-142-5p potentially targets the 3’ untranslated Region (UTR) of TROAP. **G** The expression correlation of miR-142-5p and TROAP in 28 HCC cases was analyzed by quantitative real-time PCR (qRT-PCR). **H** TROAP mRNA expression in Hep3B and PLC8024 cells was tested by qRT-PCR after transfection of miR-142-5p mimics and negative control oligonucleotides. Data are represented as mean ± SEM; two-sided Student’s *t*-test; ***P < 0.001.

Disease Oncology analysis indicates that dysregulation of *TROAP* is involved in the carcinogenesis, including hepatocarcinogenesis (Fig. 7A). Gene expression data from TCGA database and Lim HY’ cohort (GSE36376) demonstrated that *TROAP* expression was dramatically upregulated in HCC tissues than that in normal liver tissues (Fig. 7B and S5A). IHC staining also showed the higher expression of *TROAP* in HCC tissue than that in corresponding non-tumor liver tissue (Fig. 7C). Moreover, the protein expression of TROAP in one immortalized human hepatocyte cell line MIHA and nine HCC cell lines was analyzed with western blotting. Results showed the relative high expression of TROAP in HCC cell lines, compared to MIHA cells (Fig. 7D). In addition, according to the clinical data from TCGA database, expression of *TROAP* gene was gradually upregulated from early-stage to advanced-stage of HCC (Fig. S5B), as well as from the well differentiated group to the poor differentiated group (Fig. S5C). Most importantly, Kaplan–Meier survival curves based on TCGA database suggested that both overall survival (OS, *P* = 0.0035) and disease-free survival (DFS, *P* = 0.0011) of the HCC patients with high *TROAP* expression were significantly shorter than those with low levels of *TROAP* expression (Fig. 7E). In addition, analyses of the gene expression and survival data of cancer patients from TCGA database, we found that the mRNA expression of *TROAP* was dramatically upregulated in many aggressive cancer tissues and cancer cell lines (Fig. S6), and increased *TROAP* was negatively associated with the poorer OS and DFS of cancer patients (Fig. S7). These findings suggest that dysregulation of *TROAP* plays a vital role in cancer progression.

### TROAP expression is regulated by miR-142-5p in HCC

To explore the mechanism of *TROAP* upregulation in HCC, TargetScan has been used to predict the miRNAs that potentially regulated the expression of *TROAP*. Results found that *miR-142-5p* potentially binds to the 3’ UTR of *TROAP* (Fig. 7F). It has been reported that *miR-142-5p* was downregulated in HCC, and overexpression of *miR-142-5p* could inhibit HCC cell growth^19,20^. To determine if *TROAP* is regulated by *miR-142-5p* in HCC, the expression levels of *TROAP* and *miR-142-5p* were analyzed in 23 HCC samples with qRT-PCR. Results showed that the expression of *TROAP* was negatively correlated with *miR-142-5p* in HCC tissues (*r* = −0.4441, *P* = 0.0005, Fig. 7G). Most importantly, transfection of human *miR-142-5p* mimics could significantly downregulate *TROAP* expression in Hep3B and PLC8024 cells (Fig. 7H), indicating that *TROAP* expression might be regulated by *miR-142-5p* in HCC.

## Discussion

**Fig. 8:**
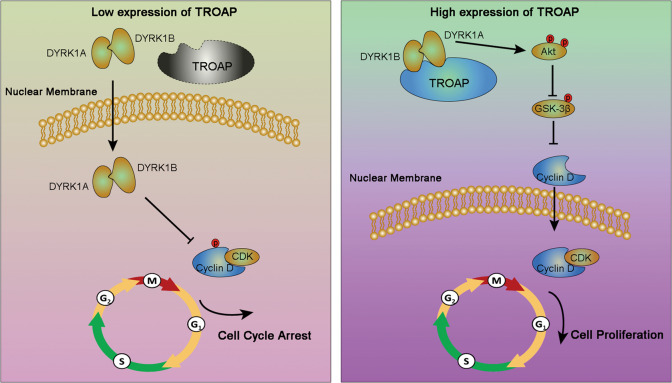
High expression of TROAP enhances HCC cell proliferation via DYRK1/Akt/GSK-3β signaling. In normal liver tissues, the expression of TROAP is downregulated by miR-142-5p. Intranuclear DYRK1A and DYRK1B induce Cyclin D1 proteolysis and cell cycle arrest. Inversely, TROAP highly expresses in HCC tissues and directly binds DYRK1A and DYRK1B, which forms a protein complex resulting in the cytoplasmic retention of DYRK1A and DYRK1B. Cytoplasmic DYRK1 activates Akt/GSK-3β signaling and enhances the stability and nuclear localization of Cyclin D1, which accelerates cell cycle process and promotes the malignant proliferation of HCC cell.

Cytoplasmic protein TROAP has been reported to be required for spindle assembly and cell division^3,21^. Recently, increasing evidences showed that dysregulation of TROAP was involved in progression of various types of cancer^6,9,10,22^. These superficial studies revealed that TROAP expression was upregulated in cancer tissues, predicting the worse outcome of cancer patients. The main functions of TROAP were revealed to promote the proliferation and metastasis of cancer cells^6,22^. However, the molecular mechanisms about the expression regulation and biological function of TROAP in HCC have yet to be fully revealed. In the present study, we confirmed the high expression of *TROAP* in HCC by analyzing TCGA dataset, which showed that dysregulation of *TROAP* was specifically regulated by *miR-142-5p*. Moreover, cytoplasmic TROAP directly bound to DYRK1, which inhibited the nuclear localization of DYRK1 activating Akt/GSK-3K-3nhibited (Fig. 8). In mouse, we also found that targeting DYRK1 could minish tumor growth for HCC cells with high expression of *TROAP*. Therefore, on the bases of previous studies, we clearly revealed the secrets of TROAP in promotion of HCC progression and preliminarily explored its therapeutic potential in HCC.

By analyzing TCGA database, we characterized that *TROAP* was significantly high expressed in 23 kinds of cancers, including HCC. Upregulated *TROAP* predicts the poor OS and DFS in patients with adrenocortical carcinoma, kidney renal clear cell carcinoma, pancreatic adenocarcinoma, skin cutaneous melanoma and HCC. The negative correlations between dysregulation of *TROAP* and prognosis of cancer patients have been investigated in many other cancers, such as prostate cancer^5^, gastric cancer^6^, breast cancer^22^ and lung adenocarcinoma^23^. However, Lian et al.^10^ reported that TROAP was downregulated in human HCC tissues, as well as cell lines at mRNA and protein levels. They further constructed *TROAP*-depletion or overexpressed HCC cells and showed that *TROAP* suppressed cellular growth and migration in HCC. Inversely, other studies and our findings strongly verified the oncogenic role of *TROAP* in HCC progression^8,9^. Research revealed that TROAP expression peaks in the cell during the G2/M phase and abruptly declines after the cell division^8^. In our study, in vitro and in vivo functional assays clearly demonstrated that overexpression of *TROAP* enhanced HCC cell proliferation by accelerating cell cycle process. The inconsistency to the above researches may be due to the different origins of HCC samples and the distinct cell lines used in these studies^10^. In addition, we revealed that the mRNA level of *TROAP* could be regulated by *miR-142-5p* in HCC cells, which suggested the complexity of TROAP in expression regulation during caner progression. Therefore, further research is needed to clarify this discrepancy.

Gene Ontology analysis and previous studies showed that *TROAP* was required to chromosome assembly and segregation, and cell cycle regulation^3,24^. Consistent with it, our IF staining in HCC cells showed that the protein expression of TROAP was significantly increased during mitosis. The G1/S phase arrest induced by TROAP silence was also detected in HCC cells. At present, there are still few researches on this mechanism. Ye et al.^5^ revealed that TROAP regulated prostate cancer progression via Wnt3/survivin pathway. By analyzing protein-protein interaction database InAct and performing IF co-localization and protein immunoprecipitation analyses, we found that DYRK1A and DYRK1B were directly bound to TROAP in cytoplasm and mediated the oncogenic function of TROAP in HCC. DYRK1 family members as serine/threonine kinases were involved in the regulation of cancer progression and cell proliferation^25–27^. DYRK1A inhibition promoted EGFR degradation in glioblastoma cells, which sharply reduced the self-renewal and proliferation of cancer cells^17^. High expression of DYRK1B was associated with a worse prognosis for patients with liposarcoma. Targeting DYRK1B with its inhibitor AZ191 reduced liposarcoma cell growth and motility^16^. Herein, RNA interference and AZ191 treatment were applied to treat HCC cells with or without *TROAP* overexpression in vitro. Results showed that blocking DYRK1 significantly attenuated the proliferation promotion of TROAP in HCC cells, which suggested vital role of DYRK1 in TROAP-mediated HCC progression.

AZ191 is a novel small-molecule DYRK1 inhibitor and exhibits 10-fold selectivity for DYRK1B over DYRK1A in cells^28^. We proved that AZ191 treatment could inhibit HCC cell growth in concentration dependent manner. However, the proliferation inhibition of AZ191 in HCC cells with low expression of *TROAP* was weaker than that in *TROAP*-overexpressed HCC cells. This data suggested that TROAP regulated the biological function of DYRK1 in cancer cells. Our further study revealed that the protein level of TROAP was associated with the cellular localization of DYRK1. Upregulated TROAP resulted in the cytoplasmic accumulation of DYRK1. Pharmacological inhibition of DYRK1B could downregulated Akt phosphorylation at Ser473 and Thr308 in human pancreatic and ovarian cancer cells^18^. Increased DYRK1B kinase resulted in the inhibition of GSK-3β through phosphorylation of Ser9 in mouse embryonic fibroblasts^29^. These evidences strongly supported our findings in HCC. We revealed that cytoplasmic DYRK1 induced by *TROAP* overexpression could also increase the phosphorylation levels of Akt^Ser473/Thr308^ and GSK-3β^Ser9^ in HCC cells. GSK-3β could cause cell arrest in G1/G0 by destabilizing Cyclin D1^30^. Therefore, TROAP interacted with DYRK1 enhancing HCC cell proliferation by Akt/GSK-3β/Cyclin D1 pathway (Fig. 8, *right*). However, recent studies showed that *DYRK1B* expression was elevated in quiescent pancreatic cancer cells, and overexpression of *DYRK1B* induced the G1/S phase arrest via phosphorylation at Thr286 and proteasome-dependent turnover of Cyclin D1^31,32^. This discrepancy may be due to the cellular localization of DYRK1 in cancer cells. We demonstrated that DYRK1 mainly located in the nucleus in HCC cells with low expression of *TROAP*. Nuclear DYRK1 promoted the phosphorylation and protein degradation of Cyclin D1 and induced the cell cycle arrest (Fig. 8, *left*). Finally, we confirmed that blocking DYRK1 with AZ191 could inhibit tumor growth for *TROAP*-overexpressed HCC cells in mouse. Moreover, AZ191 treatment enhanced the cytotoxic effect of chemotherapy drug cisplatin in vivo, which was consistent with previous studies in ovarian cancer^33,34^. Therefore, targeting DYRK1 may be a promising therapeutic treatment for HCC patients with high expression of TROAP.

Taken together, this study investigated the expression regulation, oncogenic role, functional mechanism and therapeutic potential of *TROAP* in HCC. Aberrant elevated *TROAP* drove HCC cell growth through DYRK1/Akt/GSK-3β signaling. Meanwhile, combination treatment with DYRK1 blocking and chemotherapeutic drug effectively inhibited the growth of *TROAP*-overexpressed HCC cells in mouse, which presented a novel therapeutic approach in HCC.

## Supplementary information

Supplementary Figure Legends

Figure S1

Figure S2

Figure S3

Figure S4

Figure S5

Figure S6

Figure S7

Table S1

Table S2

Table S3
