# Supplementary material for: TROAP switches DYRK1 activity to drive hepatocellular carcinoma progression
Source: Cell Death Dis. 2021 Jan 26;12(1):125. doi: 10.1038/s41419-021-03422-3 (PMC7838256; doi:10.1038/s41419-021-03422-3)
Supplement: Supplementary file 1 — Supplementary Figure Legends [file 41419_2021_3422_MOESM1_ESM.docx]

**Supplementary Figure Legends**

**Figure S1. IF double staining with antibodies against Ki67 (green) and TROAP (red) showed the high expression of TROAP during mitotic phase in HCC cells.** Cell nuclei were stained with DAPI (blue). Scale bar, 10 μm.

**Figure S2. Co-localization of TROAP and DYRK1A or DYRK1B with IF double staining in PLC8024 cells. (A)** IF staining with antibodies against TROAP (red) and DYRK1A or DYRK1B (green) in PLC8024 cells. Cell nuclei were stained with DAPI (blue). Scale bar, 10 μm. **(B)** Representative co-localization signals indicated by white line in panel **A** were analyzed with ImageJ software. **(C)** The correlation of fluorescence values between TROAP and DYRK1A or DYRK1B were analyzed, respectively.

**Figure S3. The mRNA expressions of DYRK1A and DYRK1B in vector or TROAP-transfected Huh7 cells were analyzed with qRT-PCR.** Data were indicated as mean ± SEM; two-sided Student’s *t*-test; ***, *P* < 0.001.

**Figure S4. Cell growth assay of Hep3B and PLC8024 cells treated with different concentrations of AZ191.** Data were indicated as mean ± SD; two-sided Student’s *t*-test; *, *P* < 0.05; **, *P* < 0.01; ***, *P* < 0.001.

**Figure S5. High expression of TROAP was involved in HCC progression. (A)** Gene Expression Omnibus (GEO) public dataset analysis showed the high expression of *TROAP* in HCC tissues than that in normal liver tissues (Lim HY’ cohort, GSE36376). **(B-C)** *TROAP* gene expression levels were correlated with the advanced-stage **(B)** and poor differentiation **(C)** of HCC based on TCGA database.

**Figure S6. The mRNA level of TROAP in cancer tissues and cancer cell lines.**

**(A)** TCGA database analysis showed that *TROAP* was significantly up-regulated in 22 cancers compared to normal tissues. **(B)** Most cancer cells highly expressed *TROAP* analyzed with Cancer Cell Line Encyclopedia database (https://portals.broadinstitute.org/ccle).

**Figure S7. High expression of TROAP was associated with the poor overall survival (A) and disease-free survival (B) of cancer patients.**
