# Supplementary figures and images for: TROAP switches DYRK1 activity to drive hepatocellular carcinoma progression
Source: Cell Death Dis. 2021 Jan 26;12(1):125. doi: 10.1038/s41419-021-03422-3 (PMC7838256; doi:10.1038/s41419-021-03422-3)

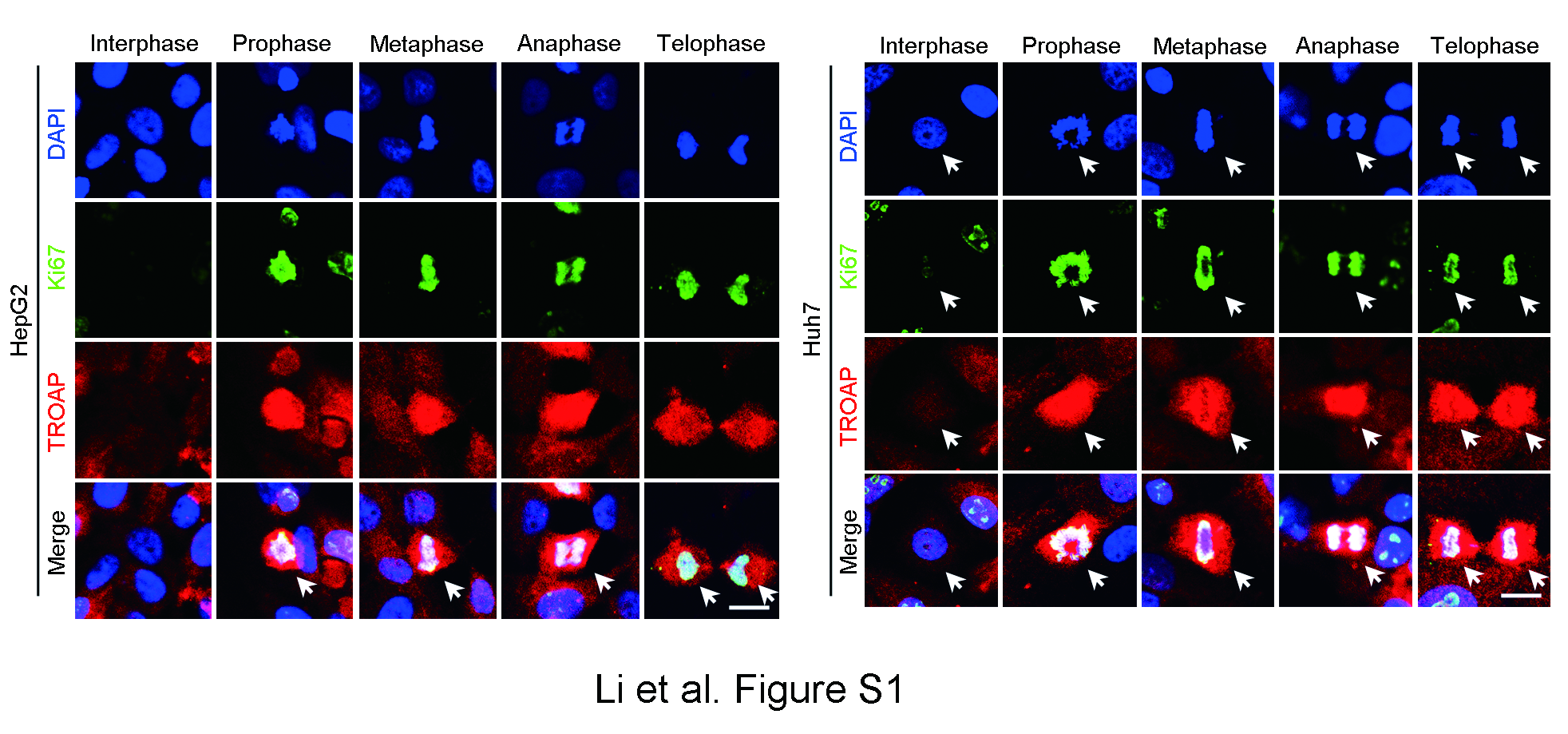

Supplement: Supplementary file 2 — Figure S1 [file 41419_2021_3422_MOESM2_ESM.tif]

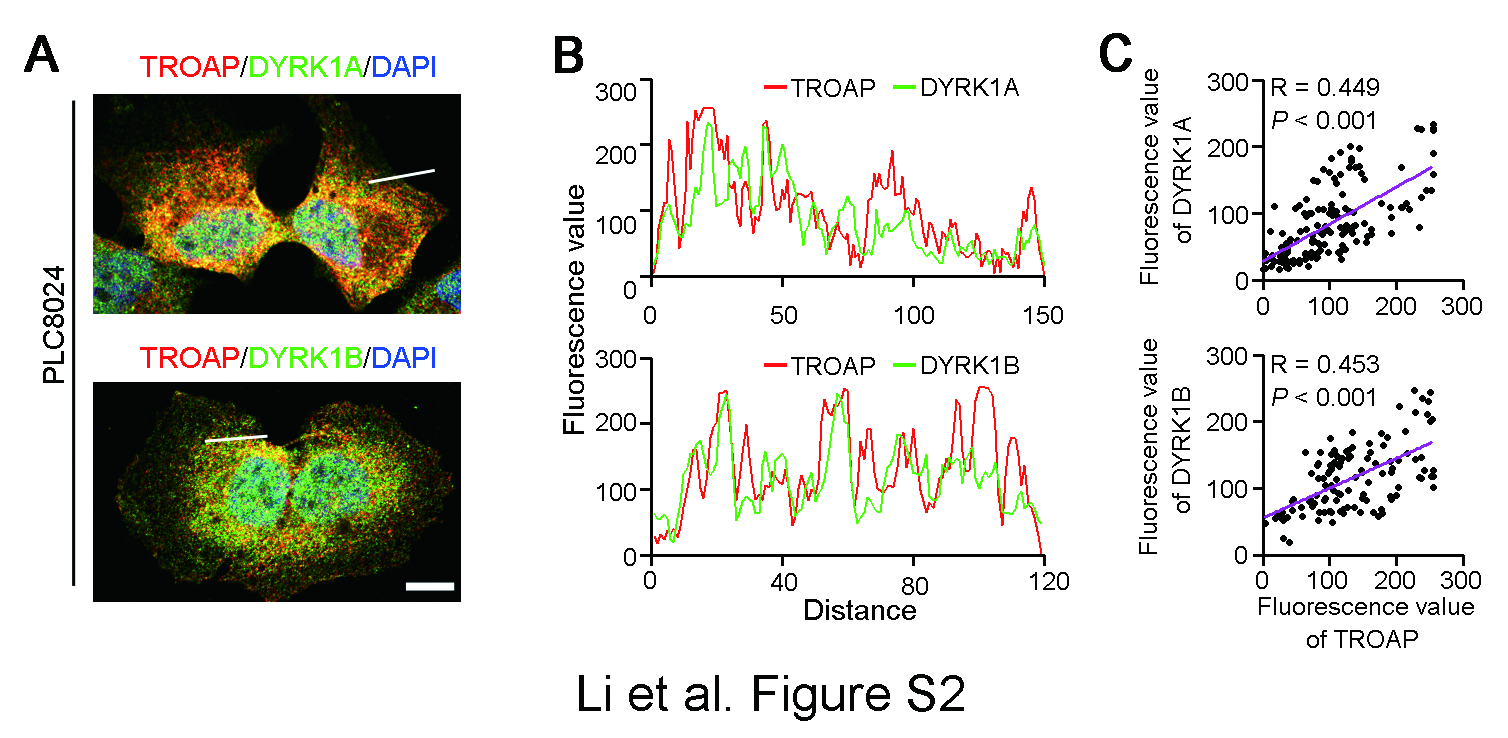

Supplement: Supplementary file 3 — Figure S2 [file 41419_2021_3422_MOESM3_ESM.tif]

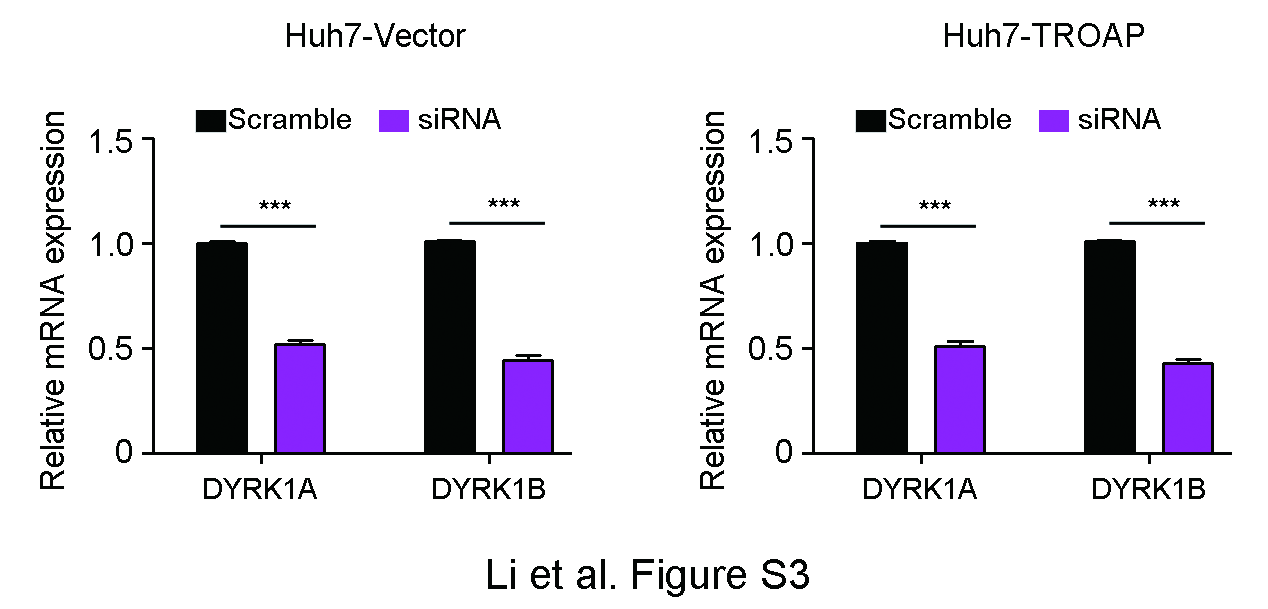

Supplement: Supplementary file 4 — Figure S3 [file 41419_2021_3422_MOESM4_ESM.tif]

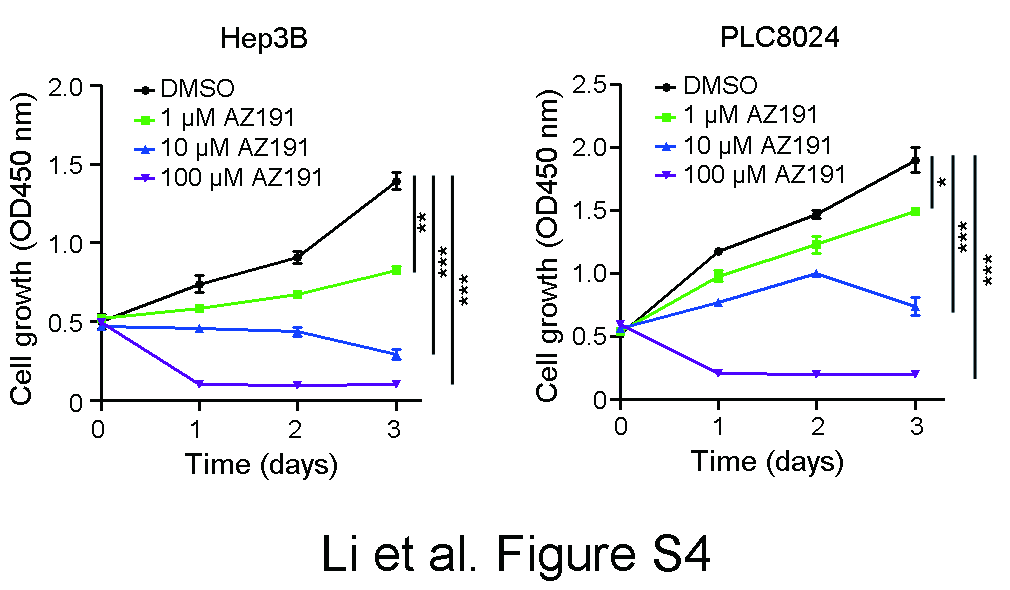

Supplement: Supplementary file 5 — Figure S4 [file 41419_2021_3422_MOESM5_ESM.tif]

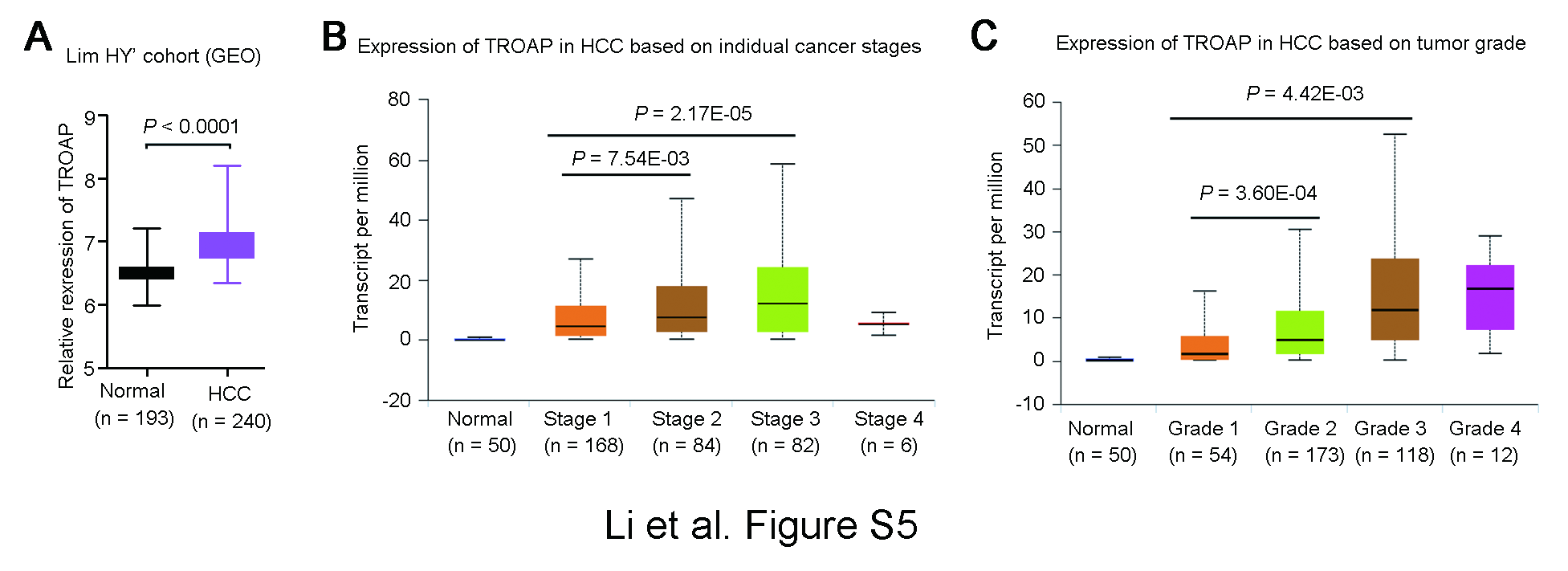

Supplement: Supplementary file 6 — Figure S5 [file 41419_2021_3422_MOESM6_ESM.tif]

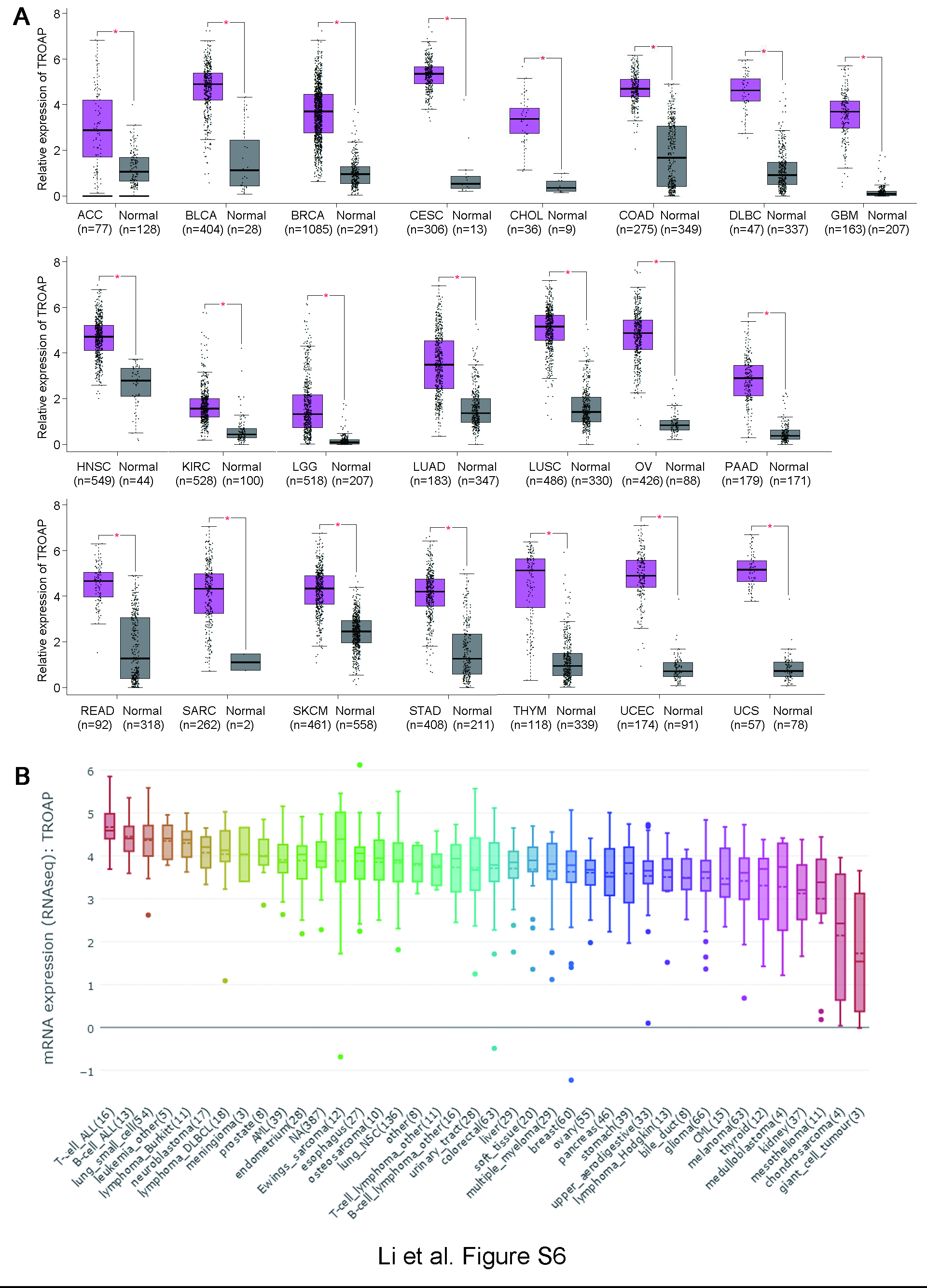

Supplement: Supplementary file 7 — Figure S6 [file 41419_2021_3422_MOESM7_ESM.tif]

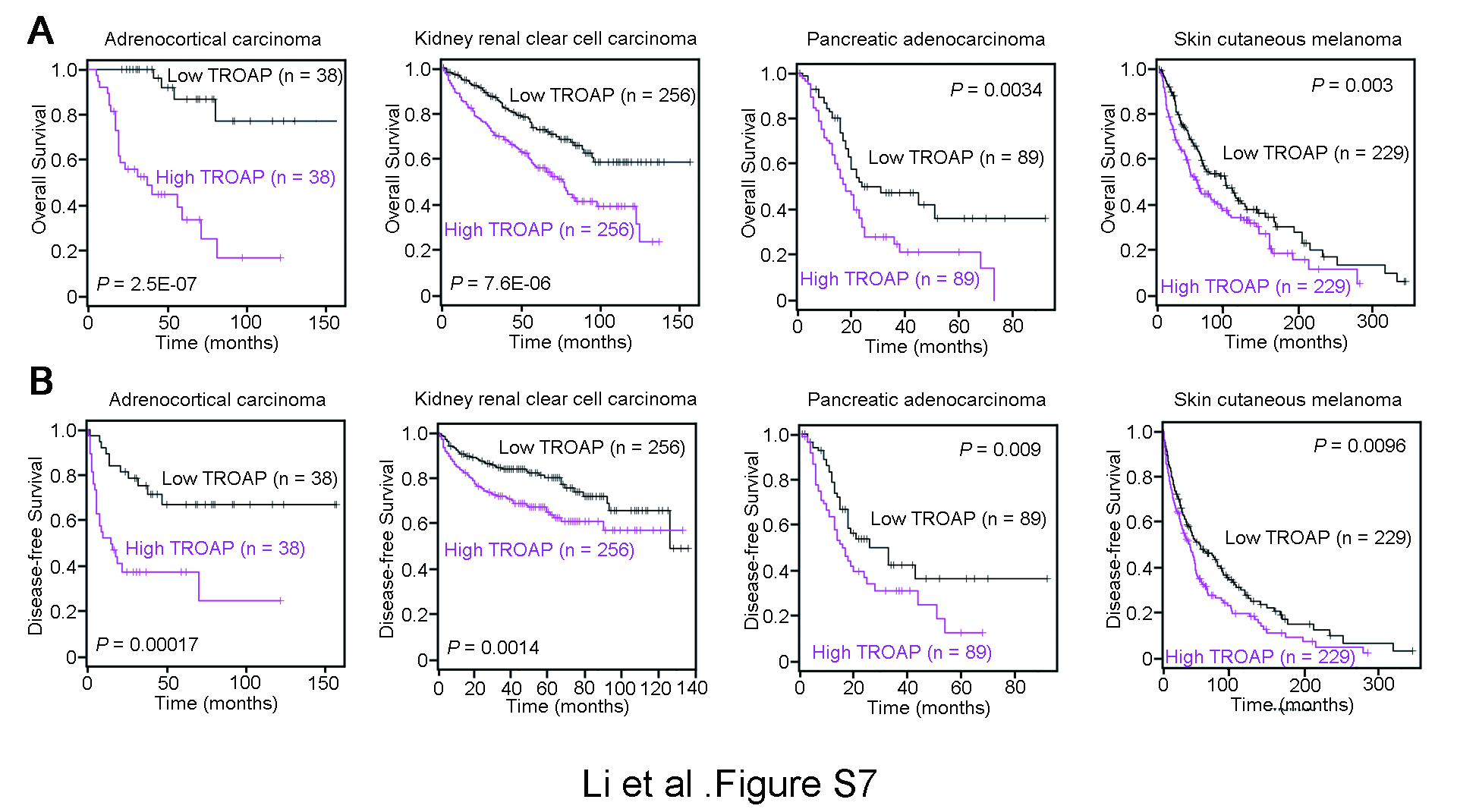

Supplement: Supplementary file 8 — Figure S7 [file 41419_2021_3422_MOESM8_ESM.tif]
