# Supplementary material for: TROAP switches DYRK1 activity to drive hepatocellular carcinoma progression
Source: Cell Death Dis. 2021 Jan 26;12(1):125. doi: 10.1038/s41419-021-03422-3 (PMC7838256; doi:10.1038/s41419-021-03422-3)
Supplement: Supplementary file 9 — Table S1 [file 41419_2021_3422_MOESM9_ESM.docx]

| **Oligonucleotides** | **Nucleotide sequence (5'-3')** |
| --- | --- |
| **RNA oligo** |  |
| Negative control | UUCUCCGAACGUGUCACGUTT |
| Hsa-miR-142-5p mimics | CAUAAAGUAGAAAGCACUACU |
|  |  |
| **siRNA** |  |
| Scramble control | GCUUCGCGCCGUAGUCUUA |
| siTROAP (shTROAP) | GGAGAGUUGUAUAAGGUCACU |
| siDYRK1A | GCGGAAGGUUUACAAUGAU |
| siDYRK1B | CGAAAGAACUCAGGAAGGA |
|  |  |
| **Primer** |  |
| miR-142-5p | (#HmiRQP0185, GeneCopoeia) (forward) |
|  | (#QP015, GeneCopoeia) (reverse) |
| U6 | CTCGCTTCGGCAGAC (forward) |
|  | AACGCTTACGAATTT (reverse) |
| TROAP | GGCAGGCCTCAGCAATCTG (forward) |
|  | GGCATCCTGCCATTCGAGTA (reverse) |
| DYRK1A | GACCAAAGATGGAAAACGGGA (forward) |
|  | CCTCCTGTTTCCACTCCAAGAA (reverse) |
| DYRK1B | ATTCACTGCGACCTCAAG (forward) |
|  | GCGGCTCTGGATATACTG (reverse) |
| ACTB | CATGTACGTTGCTATCCAGGC (forward) |
|  | CTCCTTAATGTCACGCACGAT (reverse) |

**Table S1. Oligonucleotides used in research**
