# Supplementary material for: TROAP switches DYRK1 activity to drive hepatocellular carcinoma progression
Source: Cell Death Dis. 2021 Jan 26;12(1):125. doi: 10.1038/s41419-021-03422-3 (PMC7838256; doi:10.1038/s41419-021-03422-3)
Supplement: Supplementary file 10 — Table S2 [file 41419_2021_3422_MOESM10_ESM.docx]

| **Table S2. Primary antibodies that used in western blot analysis** | | | | |
| --- | --- | --- | --- | --- |
| **Antibodies** | **Corporations** | **Catalog** | **Dilutions** |  |
| Akt | Cell Signaling Technology | #4691 | 1:2,000 |  |
| CDC2 | Cell Signaling Technology | #9112 | 1:1,000 |  |
| CDK2 | Cell Signaling Technology | #2546 | 1:1,000 |  |
| CDK4 | Cell Signaling Technology | #2906 | 1:2,000 |  |
| CDK6 | Cell Signaling Technology | #3136 | 1:2,000 |  |
| Cyclin A2 | Cell Signaling Technology | #4656 | 1:2,000 |  |
| Cyclin B1 | Cell Signaling Technology | #4135 | 1:2,000 |  |
| Cyclin D1 | Cell Signaling Technology | #2922 | 1:1,000 |  |
| Cyclin E1 | Cell Signaling Technology | #4129 | 1:1,000 |  |
| DYRK1A | Cell Signaling Technology | #8765 | 1:1,000 |  |
| DYRK1B | Cell Signaling Technology | #5672 | 1:1,000 |  |
| GAPDH | Cell Signaling Technology | #5174 | 1:3,000 |  |
| GSK-3β | Cell Signaling Technology | #12456 | 1:1,000 |  |
| Histone H3 | Cell Signaling Technology | #9717 | 1:1,000 |  |
| p-Akt(Ser473) | Cell Signaling Technology | #4060 | 1:1,000 |  |
| p-Akt(Thr308) | Cell Signaling Technology | #2965 | 1:1,000 |  |
| p-GSK-3(Ser136) | Cell Signaling Technology | #9323 | 1:1,000 |  |
| TROAP | Santa Cruz Biotechnology | #SC271716 | 1:1,000 |  |
| β-Tubulin | Cell Signaling Technology | #2128 | 1:2,000 |  |
