# Supplementary material for: TROAP switches DYRK1 activity to drive hepatocellular carcinoma progression
Source: Cell Death Dis. 2021 Jan 26;12(1):125. doi: 10.1038/s41419-021-03422-3 (PMC7838256; doi:10.1038/s41419-021-03422-3)
Supplement: Supplementary file 11 — Table S3 [file 41419_2021_3422_MOESM11_ESM.docx]

**Table S3. Proteins interacted with TROAP from IntAct database**

| **ID** | **UniProtKB** | **Proteins** | **Two Hybrid** | **Co-IP** | **BiFC** | **TAP** | **BioID** |
| --- | --- | --- | --- | --- | --- | --- | --- |
| 1 | Q9Y463 | **DYRK1B** | √ | √ | √ | √ |  |
| 2 | Q13627 | **DYRK1A** | √ |  | √ | √ |  |
| 3 | Q8N9N5 | BANP | √ | √ |  |  |  |
| 4 | P61962 | DCAF7 |  | √ |  | √ |  |
| 5 | Q15691 | MAPRE1 | √ | √ |  |  |  |
| 6 | Q5BKX5 | C19orf54 | √ |  |  |  |  |
| 7 | Q15303 | ERBB4 |  | √ |  |  |  |
| 8 | O75420 | GIGYF1 | √ |  |  |  |  |
| 9 | Q15323 | KRT31 | √ |  |  |  |  |
| 10 | Q6A162 | KRT40 | √ |  |  |  |  |
| 11 | P0DPK4 | NOTCH2NLC | √ |  |  |  |  |
| 12 | Q9UPY8 | MAPRE3 | √ |  |  |  |  |
| 13 | O43482 | OIP5 | √ |  |  |  |  |
| 14 | Q05BL1 | TP53BP2 | √ |  |  |  |  |
| 15 | Q12933 | TRAF2 | √ |  |  |  |  |
| 16 | P36406 | TRIM23 | √ |  |  |  |  |
| 17 | Q7TME2 | Spag5 |  |  |  | √ |  |
| 18 | Q12815 | TROAP | √ |  |  |  |  |
| 19 | Q5JR59-3 | MTUS2 | √ |  |  |  |  |
| 20 | G3X972 | Sec24c |  | √ |  |  |  |
| 21 | P09093 | CELA3A |  | √ |  |  |  |
| 22 | P49356 | FNTB |  | √ |  |  |  |
| 23 | P51687 | SUOX |  | √ |  |  |  |
| 24 | Q00610 | CLTC |  | √ |  |  |  |
| 25 | Q06190 | PPP2R3A |  | √ |  |  |  |
| 26 | Q13042-2 | CDC16 |  | √ |  |  |  |
| 27 | Q5SZD1 | C6orf141 |  | √ |  |  |  |
| 28 | Q6UWN0-2 | LYPD4 |  | √ |  |  |  |
| 29 | Q8IV13 | CCNJL |  |  |  |  | √ |
| 30 | Q8N0Z3 | SPICE1 |  | √ |  |  |  |
| 31 | Q8N6T7 | SIRT6 |  | √ |  |  |  |
| 32 | Q9BRK4 | LZTS2 |  | √ |  |  |  |
| 33 | Q9H6Z9 | EGLN3 |  | √ |  |  |  |
| 34 | Q9Y2I6 | NINL |  |  |  |  | √ |
| 35 | Q9Y575-2 | ASB3 |  | √ |  |  |  |

Note of interaction detection methods: Co-IP, Co-immunoprecipitation; BiFC, bimolecular fluorescence complementation; TAP, tandem affinity purification; BioID, proximity-dependent biotin identification.
